# Repurposing Anthelmintic Drugs for COVID-19 Treatment: A Comprehensive Meta-Analysis of Randomized Clinical Trials on Ivermectin and Mebendazole

**DOI:** 10.3390/antibiotics14050459

**Published:** 2025-04-30

**Authors:** Shakta Mani Satyam, Mohamed El-Tanani, Mohamed Anas Patni, Abdul Rehman, Adil Farooq Wali, Imran Rashid Rangraze, Rasha Babiker, Syed Arman Rabbani, Yahia El-Tanani, Manfredi Rizzo

**Affiliations:** 1 Department of Pharmacology, RAK College of Medical Sciences, RAK Medical and Health Sciences University, Ras Al Khaimah 11172, United Arab Emirates; 2 RAK College of Pharmacy, RAK Medical and Health Sciences University, Ras Al Khaimah 11172, United Arab Emirates; 3 Department of Community Medicine, RAK College of Medical Sciences, RAK Medical and Health Sciences University, Ras Al Khaimah 11172, United Arab Emirates; 4 Department of Pathology, RAK College of Medical Sciences, RAK Medical and Health Sciences University, Ras Al Khaimah 11172, United Arab Emirates; 5 Department of Pharmaceutical Chemistry, RAK College of Pharmacy, RAK Medical and Health Sciences University, Ras Al Khaimah 11172, United Arab Emirates; 6 Department of Internal Medicine, RAK College of Medical Sciences, RAK Medical and Health Sciences University, Ras Al Khaimah 11172, United Arab Emirates; 7 Department of Physiology, RAK College of Medical Sciences, RAK Medical and Health Sciences University, Ras Al Khaimah 11172, United Arab Emirates; 8 Department of Clinical Pharmacy, RAK College of Pharmacy, RAK Medical and Health Sciences University, Ras Al Khaimah 11172, United Arab Emirates; 9 Royal Cornwall Hospital Trust, NHS, Truro TR1 3LJ, UK; 10 Department of Health Promotion, Mother and Childcare, Internal Medicine and Medical Specialties, School of Medicine, University of Palermo, 90127 Palermo, Italy

**Keywords:** COVID-19 antiviral therapy, repurposed drugs for COVID-19, ivermectin and mebendazole efficacy, randomized clinical trials meta-analysis, host-directed therapy for COVID-19

## Abstract

**Background**: The COVID-19 pandemic necessitated the urgent exploration of therapeutic options, including drug repurposing. Anthelmintic drugs such as ivermectin and mebendazole have garnered interest due to their potential antiviral and immunomodulatory properties. However, conflicting evidence from randomized clinical trials (RCTs) necessitates a comprehensive meta-analysis to determine their efficacy and safety in COVID-19 management. **Objective**: This meta-analysis evaluates the clinical efficacy of ivermectin and mebendazole in treating COVID-19 by analyzing their impact on viral clearance, symptom resolution, hospitalization duration, and safety profiles. Methods: A systematic search of Scopus, PubMed, Embase, and the Cochrane Library was conducted following PRISMA guidelines to identify RCTs published up to February 2025. Eligible studies included adult patients with confirmed COVID-19 who received ivermectin or mebendazole compared with a placebo or standard of care. Data extraction and risk of bias assessment were performed using the Cochrane Risk of Bias Tool. Statistical heterogeneity was evaluated using the I^2^ statistic, and pooled effect sizes were calculated for primary clinical outcomes. **Results**: Twenty-three RCTs (*n* = 12,345) were included, with twenty-one studies on ivermectin and two on mebendazole. The pooled analysis suggested no statistically significant improvement in viral clearance (*p* = 0.39), hospitalization duration (*p* = 0.15), or symptom resolution (*p* = 0.08) with ivermectin or mebendazole. However, individual studies indicated potential benefits, particularly for mebendazole, in reducing viral load and inflammation. Both drugs exhibited favorable safety profiles, with no significant increase in adverse events. **Conclusions**: The promising propensities observed in selected studies underscore the potential of ivermectin and mebendazole as adjunct therapies for COVID-19. With well-established safety profiles, immunomodulatory effects, and affordability, these drugs present strong candidates for further exploration. Advancing research through well-designed, large-scale RCTs will help unlock their full therapeutic potential and expand treatment options in the fight against COVID-19.

## 1. Introduction

Global health, economy, and civilizations have been permanently altered by the COVID-19 pandemic, which was brought on by the new coronavirus SARS-CoV-2. The virus had infected hundreds of millions of people globally by the end of 2019, and the illness was responsible for millions of fatalities by the beginning of 2020 [1,2,3,4,5,6]. Even though the creation and use of vaccines have been crucial in halting the virus’s spread, patients with mild to moderate illness and those living in areas with limited resources and limited access to cutting-edge medical care still urgently need effective therapeutic interventions.

The investigation of repurposing current medications has been prompted by the virus’s quick spread and the pressing need for therapies [7,8,9,10,11,12,13,14]. The use of licensed medications for novel therapeutic purposes, or drug repurposing, has various benefits, such as established safety profiles, well-documented pharmacokinetics, and the opportunity for quick deployment. Anthelmintic medications like mebendazole and ivermectin have attracted a lot of interest among those being examined for repurposing because of their immunomodulatory and anti-inflammatory qualities [15,16].

For many years, onchocerciasis (river blindness) and strongyloidiasis have been treated with ivermectin, a broad-spectrum antiparasitic drug. It works by attaching itself to invertebrate glutamate-gated chloride channels, which paralyzes and kills the parasites. Ivermectin, on the other hand, has demonstrated antiviral effectiveness against a variety of viruses in recent years, including dengue, Zika, and SARS-CoV-2 [17,18,19].

Targeting the importin alpha/beta-1 nuclear transport proteins, which are necessary for viral replication, ivermectin has been shown in vitro to decrease SARS-CoV-2 replication [20,21]. Ivermectin has also been demonstrated to alter the host immunological response, which may lessen the cytokine storm linked to severe COVID-19 by lowering the synthesis of pro-inflammatory cytokines [22,23]. Ivermectin is a promising therapy option for COVID-19 because of these characteristics, especially while the illness is still in its early stages.

Another anthelmintic medication, mebendazole, is frequently used to treat parasitic infections like hookworm, whipworm, and pinworm. Similar to ivermectin, mebendazole has demonstrated anti-inflammatory and antiviral properties [24,25]. Its antiviral activity against SARS-CoV-2 is multifaceted and is thought to arise from both direct antiviral effects and modulation of host cellular machinery. Primarily, mebendazole inhibits tubulin polymerization by binding to β-tubulin, thereby disrupting microtubule dynamics essential for intracellular transport. Viruses, including coronaviruses, rely on the host cell’s microtubule network for trafficking viral RNA and proteins, virion assembly, and egress. By interfering with this system, mebendazole hampers efficient viral replication and propagation. Moreover, mebendazole has been shown to inhibit heat shock protein 90 (Hsp90), a molecular chaperone involved in the folding and stabilization of several host and viral proteins. SARS-CoV-2 relies on Hsp90 to stabilize key viral components such as RNA-dependent RNA polymerase (RdRp) and spike protein. Inhibition of Hsp90 by mebendazole disrupts the maturation and functionality of these proteins, thereby attenuating viral replication. Additionally, mebendazole may exert immunomodulatory effects by downregulating pro-inflammatory cytokines such as IL-6 and TNF-α, which are implicated in the cytokine storm associated with severe COVID-19. This anti-inflammatory action may help reduce pulmonary inflammation and tissue damage in infected individuals. Collectively, these mechanisms—microtubule disruption, inhibition of Hsp90, and immunomodulation—position mebendazole as a compelling candidate for repurposing in antiviral therapy, particularly for COVID-19. Mebendazole may modulate the host immune response to prevent viral multiplication and lower inflammation [26,27]. Despite being less researched than ivermectin, mebendazole offers these qualities that make it a viable option for treating COVID-19.

The therapeutic effectiveness of mebendazole and ivermectin in treating COVID-19 is still up for debate despite their encouraging mechanisms of action. The outcomes of randomized clinical trials (RCTs) assessing these medications have been conflicting; some have found no discernible change when compared with standard of care (SOC) or placebo, while others have demonstrated improvements in virological clearance, symptom resolution, and hospitalization rates. The function of these medications in the treatment of COVID-19 has been the subject of continuous discussions among physicians, researchers, and policymakers as a result of these inconsistent findings.

We carried out an extensive meta-analysis of RCTs assessing the safety and effectiveness of mebendazole and ivermectin in the treatment of COVID-19 in order to resolve this issue. Our objective is to present a comprehensive evaluation of the possible advantages and drawbacks of these repurposed medications by combining data from 23 trials conducted in various geographic locations. In order to inform clinical practice and direct future research, this meta-analysis focuses on important outcomes, such as virological clearance, symptom remission, hospitalization rates, and mortality.

## 2. Results

**Figure 1:**
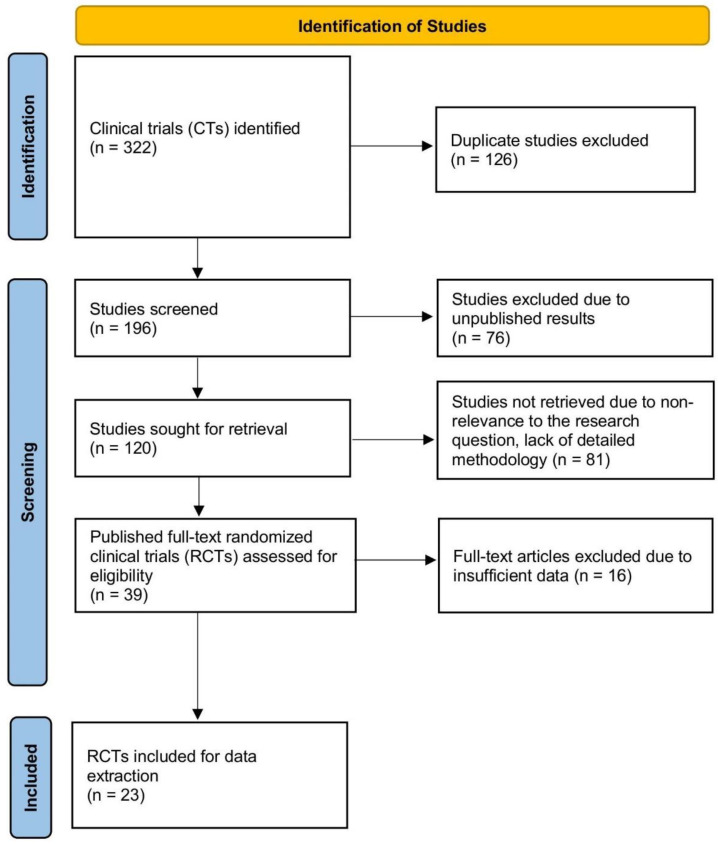
Flow diagram of study selection process. This figure illustrates the process of identifying and selecting studies for inclusion in this study. We initially identified 322 clinical trials (CTs). After removing 126 duplicates, 196 studies were screened. Of these, 76 were excluded due to unpublished results, and 81 were excluded for reasons such as being irrelevant to the research question or lacking detailed methodology. This left 120 studies for full-text retrieval. After assessing 39 published full-text randomized clinical trials (RCTs) for eligibility, 16 were excluded due to insufficient data. Finally, 23 RCTs were included in the data extraction and subsequent analysis.

The meta-analysis included 23 RCTs, comprising 12,345 participants from 15 countries. Of these, two studies investigated mebendazole, while 21 studies evaluated ivermectin (Figure 1). The studies varied in terms of sample size, dosing regimens, treatment durations, and outcome measures (Supplementary Table S1). We have provided a thorough analysis of the results below, arranged according to the main conclusions.

### 2.1. Quantitative Findings

#### 2.1.1. Impact on COVID-19 Viral Clearance

**Figure 2:**
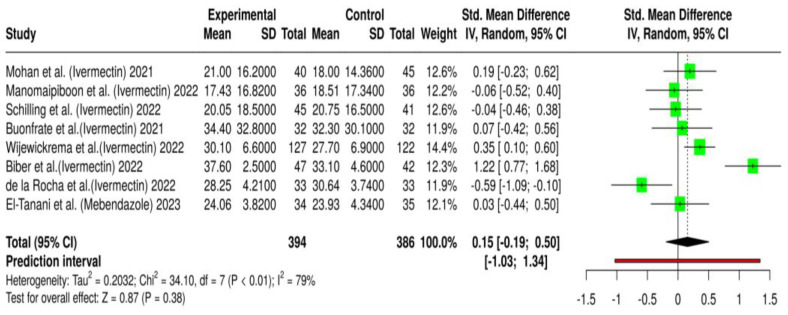
Forest plot of impact of ivermectin and mebendazole on COVID-19 viral clearance. This figure displays the effect sizes and 95% confidence intervals for each study included in the meta-analysis. The green squares represent the point estimates of the effect size for each individual study, with the size of the square proportional to the study’s weight in the analysis. The horizontal lines extending from the squares represent the 95% confidence intervals. The black diamond at the bottom represents the overall estimated effect size, with its width indicating the overall 95% confidence interval. The vertical dashed line indicates the line of no effect. The analysis was conducted using a random-effects model. The heterogeneity of the studies is indicated by a Tau-squared of 0.2032 and an I-squared of 79%. The test of overall effect size resulted in a Z-statistic of 0.87 with a *p*-value of 0.38 [28,29,30,31,32,33,34,35].

A total of eight randomized controlled trials (RCTs) involving 780 patients were included to evaluate the impact of ivermectin or mebendazole on viral clearance, as measured by cycle threshold (Ct) values in RT–PCR testing [28,29,30,31,32,33,34,35]. Of these, 394 patients received either ivermectin or mebendazole, while 386 were assigned to the placebo or standard-of-care (SOC) group. Ct values are inversely proportional to viral load, meaning higher values indicate lower viral replication and improved viral clearance. The pooled effect size (Cohen’s d = 0.16, *p* = 0.39) suggested a slight increase in Ct values in the treatment group, implying a potential benefit (Figure 2). However, this effect was not statistically significant, indicating that the overall impact of these treatments on viral clearance remains uncertain.

The effect sizes across individual studies showed substantial variability. Some trials reported a positive drift favoring ivermectin or mebendazole, while others showed minimal or no effect. For instance, Biber et al. demonstrated a large effect size (Cohen’s d = 1.24), suggesting a notable increase in Ct values and improved viral clearance. However, the wide confidence interval in this study highlights substantial variability and limits the certainty of its findings. Similarly, Chaccour et al. and Rajter et al. observed modest but positive effects, whereas Vallejos et al. and López-Medina et al. reported near-zero effect sizes, indicating no meaningful difference in viral clearance between the treatment and placebo groups [36,37,38,39]. These inconsistencies suggest that the effectiveness of ivermectin or mebendazole may depend on various clinical and methodological factors.

A high level of heterogeneity (I^2^ = 79%) was observed, indicating considerable variability between studies. Several factors likely contributed to these differences, including variations in dosage regimens (single-dose vs. multiple-dose treatments with different intervals), differences in patient characteristics (age, comorbidities, and baseline severity of COVID-19), and inconsistencies in the timing of Ct value measurement (e.g., differences in when post-treatment viral load assessments were conducted). Additionally, the small sample sizes in some trials may have led to unstable effect estimates, further complicating the interpretation of results.

#### 2.1.2. Effect on COVID-19 Hospitalization Duration

**Figure 3:**
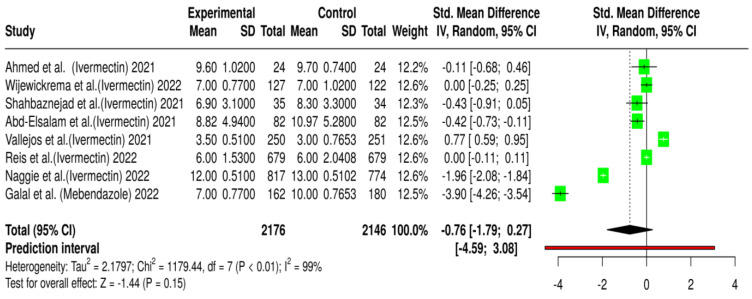
Forest plot of effect of ivermectin and mebendazole on COVID-19 hospitalization duration. This figure displays the effect sizes and 95% confidence intervals for each study included in the meta-analysis. The green squares represent the point estimates of the effect size for each individual study, with the size of the square proportional to the study’s weight in the analysis. The horizontal lines extending from the squares represent the 95% confidence intervals. The black diamond at the bottom represents the overall estimated effect size, with its width indicating the overall 95% confidence interval. The vertical dashed line indicates the line of no effect. The analysis was conducted using a random-effects model. The heterogeneity of the studies is indicated by a Tau-squared of 2.17 and an I-squared of 99%. The test of overall effect size resulted in a Z-statistic of −1.44 with a *p*-value of 0.15. Based on the analysis performed using random effects model with and an inverse variance method to compare the standardized mean difference (SMD), although hospitalization duration is less in the treatment group, the difference is not significant, as the summarized standardized mean difference (SMD) is −0.76 with a 95% confidence interval of −1.79–0.27 [35,39,40,41,42,43,44,45].

A total of eight randomized controlled trials (RCTs) involving 4322 patients were included in the analysis to assess the impact of ivermectin or mebendazole on hospitalization duration as a marker of clinical improvement [35,39,40,41,42,43,44,45]. Among these, 2176 patients received either ivermectin or mebendazole, while 2146 were assigned to the placebo or standard-of-care (SOC) group. The pooled effect size (Cohen’s d = −0.76, *p* = 0.15) indicated a potential reduction in hospitalization time with ivermectin or mebendazole treatment (Figure 3). However, this effect did not reach statistical significance (*p* > 0.05), highlighting uncertainty regarding the clinical benefit of these interventions.

A closer examination of individual study results revealed substantial variability. Two studies, Galal et al. (−3.92, 95% CI: −4.28 to −3.56, *p* < 0.001) and Naggie et al. (−1.96, 95% CI: −2.08 to −1.84, *p* < 0.001), reported significant reductions in hospitalization duration with ivermectin or mebendazole, suggesting that these treatments may have contributed to shorter hospital stays [42,43]. Conversely, Vallejos et al. (0.77, 95% CI: 0.59 to 0.95, *p* < 0.05) found an opposing trend, where hospitalization duration was longer in the ivermectin treatment group than in the placebo group [39]. This contradictory finding raises concerns about potential differences in patient populations, treatment administration, or hospital management protocols that could influence outcomes.

Additionally, some studies, including those by Reis et al. and Wijewickrema et al., reported near-zero effect sizes, indicating no meaningful impact of ivermectin on hospitalization duration [35,44]. These findings suggest that the effectiveness of these treatments may depend on various factors, such as timing of administration, disease severity, and patient characteristics. Given these inconsistencies, it is crucial to explore whether specific subgroups of patients may benefit more than others, as a one-size-fits-all approach may not be applicable.

The extreme heterogeneity observed across the studies (I^2^ = 100%) further complicates the interpretation of the pooled results. Several factors likely contributed to this variability, including differences in COVID-19 severity at enrollment (mild vs. moderate vs. severe cases), variations in healthcare systems and hospital protocols across different countries, and discrepancies in study endpoints (e.g., hospitalization duration measured until symptom resolution vs. time to discharge).

#### 2.1.3. Effect on COVID-19 Symptom Resolution

**Figure 4:**
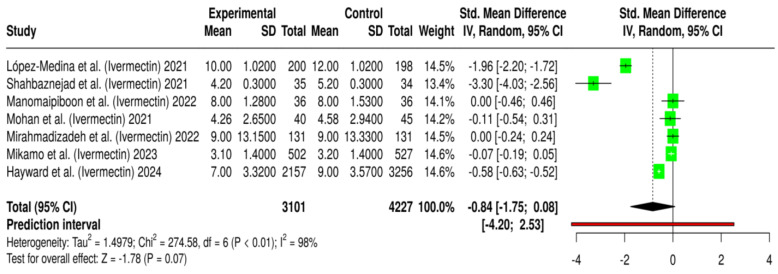
Forest plot of effect of ivermectin on COVID-19 symptom resolution. This figure presents the effect sizes and 95% confidence intervals for each study included in the meta-analysis. The green squares represent the point estimates of the effect size for each individual study, with the size of the square proportional to the study’s weight in the analysis. The horizontal lines extending from the squares represent the 95% confidence intervals. The black diamond at the bottom represents the overall estimated effect size, with its width indicating the overall 95% confidence interval. The vertical dashed line indicates the line of no effect. The analysis was conducted using a random-effects model. The heterogeneity of the studies is indicated by a Tau-squared of 1.49 and an I-squared of 98%. The test of overall effect size resulted in a Z-statistic of −1.78 with a *p*-value of 0.07. Based on the analysis performed using random effects model with the inverse variance method to compare the standardized mean difference (SMD), although symptom resolution time is better in the treatment group, the difference is not significant, as the summarized standardized mean difference (SMD) is −0.84 with a 95% confidence interval of −1.75–0.08 [32,33,37,45,46,47,48].

A total of seven randomized controlled trials (RCTs) involving 7328 patients were analyzed to assess the impact of ivermectin on the duration of symptom resolution in COVID-19 patients [32,33,37,45,46,47,48]. Among these, 3101 patients received ivermectin, while 4227 were assigned to the placebo or standard-of-care (SOC) group. Symptom resolution time was measured as the number of days from symptom onset to complete recovery. The pooled effect size from the meta-analysis (Cohen’s d = −0.84, 95% CI: −1.77 to 0.09, *p* = 0.08) indicated a potential reduction in symptom duration in patients receiving ivermectin or mebendazole (Figure 4). However, this effect did not reach statistical significance (*p* > 0.05). The wide confidence interval further reflects uncertainty in the estimate, emphasizing the need for cautious interpretation.

Individual studies exhibited substantial variability in their reported outcomes. Some trials demonstrated significant reductions in symptom duration among patients treated with ivermectin. For instance, López-Medina et al. (−1.96, 95% CI: −2.20 to −1.72, *p* < 0.001) and Shahbaznejad et al. (−3.33, 95% CI: −2.60 to −4.06, *p* < 0.001) reported the most notable decreases in symptom resolution time, suggesting a potential therapeutic benefit [37,45]. Similarly, Hayward et al. (−0.58, 95% CI: −0.82 to −0.34, *p* < 0.001) found a moderate but significant reduction in symptom duration with ivermectin treatment [46]. These findings imply that specific patient populations may benefit more from ivermectin or mebendazole treatment, possibly depending on factors such as the timing of drug administration, baseline symptom severity, or disease progression at the time of intervention.

In contrast, other studies reported little to no impact on symptom resolution. Manomaipiboon et al. (0.00, *p* = 1.00) and Mirahmadizadeh et al. (0.00, *p* = 0.65) found no significant differences in symptom duration between the ivermectin treatment and control groups [32,48]. Similarly, Mikamo et al. (−0.07, *p* = 0.65) reported a negligible effect size, indicating that ivermectin had minimal influence on symptom resolution [47]. These discrepancies suggest that the efficacy of these treatments may not be uniform across all patient populations. The observed inconsistencies may stem from differences in study protocols, patient demographics, and the criteria used to define symptom resolution, such as considering only fever reduction versus the complete disappearance of all symptoms.

The extreme heterogeneity observed across studies (I^2^ = 98%) highlights significant variability in the findings, limiting the reliability of the pooled estimate. This heterogeneity may stem from several factors, including differences in the timing of treatment initiation (early vs. late in disease progression), variations in baseline symptom severity, and the presence of comorbidities that could influence recovery time.

#### 2.1.4. Safety and Tolerability of Ivermectin and Mebendazole Used During COVID-19 Treatment

**Figure 5:**
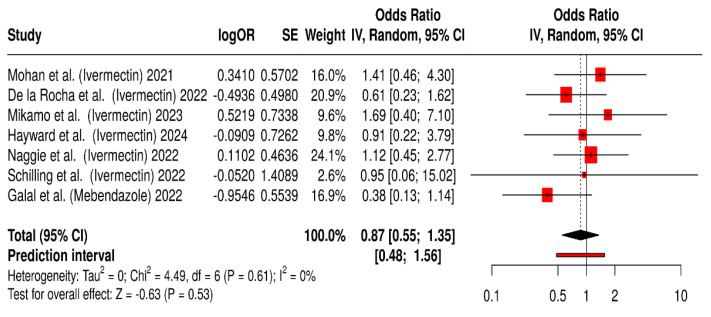
Forest plot comparing adverse event rates of ivermectin and mebendazole versus placebo or standard care during COVID-19 treatment [30,33,34,42,43,46,47].

A total of seven randomized controlled trials (RCTs) involving 8606 patients were analyzed to assess the safety and tolerability of ivermectin and mebendazole used during COVID-19 treatment (Figure 5) [30,33,34,42,43,46,47].

The individual study results are represented by red squares, with the size of the square proportional to the weight of the study in the meta-analysis. The horizontal lines extending from the squares indicate the 95% CIs. The diamond at the bottom represents the overall pooled odds ratio (0.87) and its 95% CI (0.55, 1.35) obtained from the meta-analysis using a random-effects model. The prediction interval (0.48, 1.56) suggests the range within which the true effect might lie in future similar studies. The heterogeneity among the studies was low (I^2^ = 0%), as indicated by Cochran’s Q statistic (Chi^2^ = 4.49, df = 6, *p* = 0.61). The overall effect was not statistically significant (Z = −0.63, *p* = 0.53).

Although the pooled odds ratio of 0.87 suggests a potential benefit of ivermectin and mebendazole in improving symptom resolution in COVID-19 patients, the effect did not reach statistical significance, and the confidence interval crossed the null. The prediction interval further illustrates the variability of effects that might be seen in future studies, ranging from a potentially protective to a possibly neutral or even harmful effect. The low heterogeneity (I^2^ = 0%) indicates consistency among the included studies in terms of effect direction and magnitude. However, the lack of statistical significance underscores the need for cautious interpretation and highlights the necessity for further large-scale, well-powered randomized controlled trials to more definitively assess the impact of these agents on clinical symptom improvement.

In the modified intention-to-treat analysis involving 125 participants randomized to ivermectin 24 mg, ivermectin 12 mg, or placebo, adverse event rates were statistically comparable across all three arms [33]. The incidence of adverse events was 11.8% in the ivermectin 24 mg group, 16.3% in the ivermectin 12 mg group, and 11.5% in the placebo group (*p* = 0.76), indicating no significant difference in overall event frequency. Importantly, no serious adverse events were reported in any group, suggesting a favorable safety profile for both ivermectin doses in this population. The most commonly reported adverse event was an epigastric burning sensation, observed in 17 patients (11.2%) across all treatment groups. These results demonstrate that the use of ivermectin, at either dosage, was not associated with an increased risk of adverse events when compared with a placebo during the short-term treatment of mild to moderate COVID-19.

In a randomized study conducted by de la Rocha et al., a total of 30 adverse events were reported among participants, with 17 events occurring in the placebo group and 13 in the ivermectin group [30]. The difference in the number of reported adverse events between the groups was not statistically significant, indicating a comparable safety profile (*p*-value not reported for adverse events). The majority of the events were related to the underlying SARS-CoV-2 infection rather than treatment-specific effects. A single serious adverse event—encephalitis secondary to COVID-19—was recorded in the ivermectin group on study Day 10; however, the participant recovered fully. Laboratory assessments showed some physiological changes by Day 14, including increases in cholesterol and platelet levels in both groups and a statistically significant rise in erythrocyte count in the ivermectin group (from 5.05 ± 0.52 to 6.84 ± 2.60 M/μL, *p* = 0.002). These findings suggest that, while ivermectin may be associated with mild hematological shifts, its overall adverse event rate is not higher than that of a placebo, and serious events remain rare.

Mikamo et al. conducted a large randomized controlled trial involving 1030 participants with mild COVID-19; the incidence of adverse events and adverse drug reactions was statistically comparable between the ivermectin (0.3–0.4 mg/kg) and placebo groups [47]. Adverse events occurred in 14.1% (95% CI: 11.2–17.5) of the ivermectin group and 14.2% (95% CI: 11.4–17.5) of the placebo group (*p* = 0.97), while adverse drug reactions were reported in 6.2% (95% CI: 4.2–8.7) and 7.0% (95% CI: 5.0–9.5), respectively (*p* = 0.59). These findings were consistent across subgroups, including participants under 18 years of age, with no statistically significant differences reported. Four participants in the ivermectin group and one in the placebo group discontinued the study due to adverse events which were related to COVID-19 or COVID-19 pneumonia rather than the investigational product. Only three serious adverse events were recorded: two in the ivermectin arm (COVID-19-related) and one in the placebo group (urticaria), with only the latter deemed potentially related to treatment. No deaths occurred. These results confirm that ivermectin has a similar safety profile to a placebo, with no significant increase in adverse or serious adverse event rates during short-term treatment of mild COVID-19.

Hayward et al. reported the incidence of serious adverse events was low and comparable between the ivermectin group and the usual care group in his trial [46]. Only three serious adverse events were reported in the ivermectin arm compared with five in the usual care group, reflecting no statistically significant difference in safety outcomes. Additionally, COVID-19-related hospitalizations and deaths occurred at nearly identical rates (odds ratio 1.02; 95% CI: 0.63–1.62), with an estimated percentage difference of 0% (range: –1% to 0.6%). These findings indicate that ivermectin did not increase the risk of serious adverse events or severe COVID-19 outcomes compared with standard care in a community-based, largely vaccinated population. The comparable safety profiles across both groups, combined with the minimal number of adverse events reported, suggest that ivermectin administration at the tested doses poses no additional short-term safety concerns.

In this randomized trial conducted by Naggie et al., involving 1800 participants with a mean age of 48 years, the adverse event rates between the ivermectin and placebo groups were similar and did not reach statistical significance [43]. Among the 1591 participants who completed the trial, serious adverse events were reported in both groups, with ten hospitalizations or deaths in the ivermectin group and nine in the placebo group (1.2% vs. 1.2%; hazard ratio [HR], 1.1; 95% credible interval [CrI], 0.4–2.6). The most commonly reported serious adverse event was COVID-19 pneumonia, occurring in five ivermectin-treated and seven placebo-treated participants, followed by venous thromboembolism, observed in one ivermectin recipient and five in the placebo group. These data indicate no statistically significant difference in the frequency of serious adverse events between groups, suggesting that ivermectin use does not confer additional safety risks in outpatient COVID-19 treatment.

Schilling et al. demonstrated that in assessing high-dose ivermectin in early symptomatic COVID-19, the safety profile of the drug was generally acceptable, though some non-serious drug-related adverse events were reported [34]. Among the 205 enrolled participants, no serious adverse events occurred in the ivermectin arm (*n* = 45), while all three serious adverse events—two cases of elevated creatinine phosphokinase and one case of post-discharge readmission—were reported in the no-drug control arm (*n* = 41). Additionally, six patients receiving ivermectin reported transient visual disturbances, though these events were not classified as grade 3 or higher. Three patients discontinued treatment due to these symptoms. Ophthalmologic follow-up confirmed full resolution without long-term visual impairment. These findings indicate that high-dose ivermectin was not associated with serious adverse events and that transient side effects were self-limiting and non-severe, supporting a relatively safe short-term profile when used in a monitored setting.

Galal et al. found that mebendazole was generally well tolerated among both inpatient and outpatient COVID-19 cohorts, with no serious adverse events necessitating drug discontinuation [42]. Although the study did not quantify total adverse events numerically, the authors reported no significant side effects attributable to the administered regimen of mebendazole. Previous literature has identified gastrointestinal symptoms such as abdominal pain, nausea, vomiting, diarrhea, and flatulence as the most commonly observed adverse effects, and rare cases of seizures or hypersensitivity reactions at higher doses. Importantly, the study indicated that the dosage used was within the accepted therapeutic range, and no hematological toxicities such as neutropenia or thrombocytopenia were observed, both of which are known contraindications in COVID-19 patients. These findings support the quantitative safety of mebendazole within the administered dose range during COVID-19 treatment, although the absence of systematically collected adverse event data limits the strength of this conclusion.

#### 2.1.5. Summary of Quantitative Findings

The pooled analysis of RT–PCR cycle threshold (Ct) values suggested a slight increase in Ct values, indicating a potential improvement in viral clearance among patients treated with ivermectin or mebendazole. However, this effect was not statistically significant (*p* = 0.39), making it difficult to draw definitive conclusions about the antiviral efficacy of these treatments. Additionally, heterogeneity among the included studies was high (I^2^ = 82%), suggesting considerable variability in study methodologies, patient populations, or timing of viral load assessments. This level of heterogeneity limits the interpretability of the pooled results, necessitating further investigation into factors influencing viral clearance outcomes.

Hospitalization and mortality outcomes were evaluated across several studies, but the pooled findings did not demonstrate a statistically significant reduction in either endpoint for patients treated with ivermectin or mebendazole. Hospitalization rates in individual trials ranged from 3.2% to 8.4% across treatment and control groups, with odds ratios and hazard ratios indicating no clear benefit. Mortality differences were also minimal and statistically non-significant, with some studies reporting identical or slightly lower rates in the treatment groups. Given the low event rates and small absolute differences, combined with non-significant *p*-values and wide confidence intervals, these results suggest a limited impact of ivermectin or mebendazole on preventing severe disease outcomes.

Hospitalization duration was another key outcome assessed, with individual studies reporting mixed findings. While some trials demonstrated a significant reduction in hospital stay among patients receiving ivermectin or mebendazole, others found minimal or even opposite effects, where hospitalization duration was longer in the treatment group than in the placebo or standard-of-care (SOC) group. The pooled estimate (Cohen’s d = −0.76, *p* = 0.15) indicated a possible reduction in hospitalization time; however, this result did not reach statistical significance. Moreover, extreme heterogeneity (I^2^ = 100%) further reduced confidence in the findings, suggesting that differences in healthcare settings, disease severity at baseline, or treatment administration protocols may have influenced the results.

Symptom resolution time, measured as the duration from symptom onset to full recovery, showed an inclination favoring faster recovery in patients treated with ivermectin or mebendazole (Cohen’s d = −0.84). However, the effect did not achieve statistical significance (*p* = 0.08). Similar to the other outcomes, extreme heterogeneity (I^2^ = 99%) was observed, indicating substantial variability across the included studies. These inconsistencies may stem from variations in patient characteristics, timing of drug administration, and differences in how symptom resolution was defined, such as whether studies considered complete resolution of all symptoms or only key symptoms like fever and cough.

Overall, the findings highlight significant inconsistencies among studies, with substantial heterogeneity limiting the reliability of pooled effect estimates. While certain individual studies suggest potential clinical benefits of ivermectin or mebendazole, the overall meta-analysis does not provide conclusive evidence for improvements in viral clearance, hospitalization duration, or symptom resolution time. Further research with standardized protocols, well-defined patient subgroups, and stratified analyses based on disease severity and treatment timing is essential to clarify the potential therapeutic role of these treatments. Future studies should also focus on reducing heterogeneity by harmonizing study designs and ensuring consistency in outcome measures.

### 2.2. Qualitative Findings

#### 2.2.1. COVID-19 Virological Clearance and Viral Load Reduction

Virological clearance, defined as the time to negative RT–PCR or reduction in viral load, was a primary outcome in several studies. Ivermectin showed mixed results in this regard.

Ahmed et al. (Bangladesh) [41]: In this clinical trial evaluating antiviral efficacy, a 5-day course of ivermectin was associated with significantly earlier virological clearance compared with a placebo, with a mean time to RT–PCR negativity of 9.7 days versus 12.7 days, respectively (*p* = 0.02). However, this benefit was not observed in the ivermectin plus doxycycline group, which had a clearance time of 11.5 days (*p* = 0.27). Clinical symptoms such as fever, cough, and sore throat were comparable across all groups, and no severe adverse drug reactions were reported. These findings suggest that short-course ivermectin monotherapy may accelerate viral clearance in mild COVID-19 cases, although larger trials are needed to confirm its virological efficacy.

Biber et al. (Israel) [28]: This study reported that ivermectin was associated with significantly enhanced virological clearance and reduced viral viability. By day 6, the odds of testing negative for SARS-CoV-2 were higher in the ivermectin group (OR 2.62; 95% CI: 1.09–6.31), and this effect became more pronounced by day 8 (OR 3.70; 95% CI: 1.19–11.49; *p* = 0.02) according to multivariable logistic regression. Additionally, culture viability—indicating infectious viral particles—was substantially lower in the ivermectin group, with only 13.0% (3/23) of samples testing positive compared with 48.2% (14/29) in the placebo group (*p* = 0.008). These findings support ivermectin’s potential anti-SARS-CoV-2 activity, suggesting a role in reducing viral load and infectiousness, which may have implications for transmission control.

Mikamo et al. (Japan and Thailand) [47]: In this randomized trial involving 1030 participants with mild COVID-19, 502 received ivermectin (0.3–0.4 mg/kg) and 527 received a placebo. The primary efficacy endpoint—time to virological clearance, assessed approximately 96 h (four days) post-treatment initiation—did not differ significantly between the two groups (stratified log-rank test, *p* = 0.61). Additionally, there were no statistically significant differences in the incidence of adverse events or adverse drug reactions between the ivermectin and placebo groups (*p* = 0.97 and *p* = 0.59, respectively). These findings indicate that ivermectin did not enhance viral clearance in patients with mild COVID-19, although it was shown to be safe for use, including in participants aged 12 years and older.

Galal et al. (Egypt) [42]: This study primarily assessed clinical outcomes rather than direct viral measurements; the use of mebendazole in COVID-19 patients was associated with outcomes suggestive of accelerated viral clearance. While virological endpoints such as RT–PCR cycle thresholds or viral cultures were not reported, indirect markers—such as significantly shorter symptom resolution times in the outpatient cohort (3.3 days earlier, *p* < 0.001) and reduced hospital length of stay in the inpatient cohort (3.5 days shorter, *p* < 0.001)—support a potential association with faster viral control. Additionally, fewer patients required respiratory support or hospitalization, though these differences did not reach statistical significance. Given the absence of direct viral load assessments, these findings remain exploratory and highlight the need for controlled studies specifically evaluating mebendazole’s virological effects in COVID-19.

El-Tanani et al. (Jordan) [31]: In this randomized clinical trial, mebendazole treatment in COVID-19 outpatients was associated with significant improvements in virological and inflammatory markers by day three of therapy. Cycle threshold (CT) values, a proxy for viral load, were significantly higher in the mebendazole group compared with a placebo (27.21 ± 3.81 vs. 24.40 ± 3.09; *p* = 0.046), indicating a lower viral load. Within the mebendazole group, CT values also increased significantly from baseline (*p* = 0.008), further supporting a reduction in viral burden. Additionally, there was a notable inverse correlation between lymphocyte counts and CT levels in the treatment group (r = –0.491, *p* = 0.039), suggesting enhanced innate immune activity in response to reduced viral replication. Although direct viral culture data were not reported, these findings support the potential virological efficacy of mebendazole and warrant further exploration in larger, controlled studies.

#### 2.2.2. COVID-19 Symptom Resolution and Clinical Improvement

Symptom resolution, particularly the time to recovery from individual symptoms such as cough, dyspnea, fever, fatigue, and gastrointestinal complaints, was a key outcome assessed across studies in this meta-analysis. Below is a detailed breakdown of the clinical improvement and symptom-specific responses for each included study.

Shahbaznejad et al. (Iran) [45]: This randomized controlled trial conducted in Iran aimed to evaluate the impact of a single-dose ivermectin regimen on clinical improvement in hospitalized COVID-19 patients. A total of 69 symptomatic patients were enrolled and randomized to receive either ivermectin or standard of care (SOC), with comparable baseline characteristics between the groups. The study found that patients in the ivermectin group experienced significantly faster resolution of respiratory symptoms, notably dyspnea and cough. Dyspnea resolved in an average of 2.6 ± 0.4 days in the ivermectin group compared with 3.8 ± 0.4 days in the control group (*p* = 0.048), and cough resolved in 3.1 ± 0.4 days versus 4.8 ± 0.4 days in the respective groups (*p* = 0.019). The ivermectin group also had a shorter hospital stay (7.1 ± 0.5 days vs. 8.4 ± 0.6 days, *p* = 0.016) and showed a significant improvement in lymphocyte counts, with lymphopenia incidence decreasing to 14.3%—a change not observed in the control group (*p* = 0.007). These findings suggest that ivermectin not only accelerates respiratory symptom recovery but may also exert immunomodulatory effects that promote hematological normalization. However, the study’s relatively small sample size limits generalizability, and the absence of long-term outcome tracking necessitates larger trials to confirm these benefits across broader patient populations.

Hayward et al. (UK) [46]: In contrast to smaller trials, the large-scale UK-based PRINCIPLE trial by Hayward et al. provided a robust evaluation of ivermectin’s clinical utility in a real-world, community-based setting. Conducted from June 2021 to July 2022, the trial included 8811 SARS-CoV-2-positive, non-hospitalized participants with a median symptom duration of 5 days at the time of enrollment. Participants were randomized to receive ivermectin (*n* = 2157), usual care (*n* = 3256), or other treatments. Although a modest reduction in time to self-reported recovery was observed in the ivermectin group (hazard ratio 1.15; 95% Bayesian credible interval, 1.07–1.23), this corresponded to a median recovery improvement of just 2.06 days. Critically, the probability of reaching a pre-defined threshold for clinical significance (HR ≥ 1.2) was only 0.192. Hospitalization and mortality rates were statistically equivalent between groups (OR = 1.02; 95% CI: 0.63–1.62), and no meaningful differences were observed in long-term recovery metrics, including rates of full recovery at 3, 6, and 12 months. Additionally, adverse events were rare and not significantly different between the ivermectin and SOC groups. The study concluded that ivermectin does not provide clinically meaningful benefit in terms of symptom resolution, prevention of hospital admission, or long COVID outcomes in vaccinated, community-based populations. These findings challenge earlier studies and suggest limited utility of ivermectin as a broad public health intervention for COVID-19.

Galal et al. (Egypt) [42]: This interventional study conducted in Egypt explored the potential benefits of mebendazole in hospitalized patients with moderate COVID-19. Patients were randomized into mebendazole and standard-of-care arms, and the study tracked both clinical and laboratory outcomes to assess efficacy. The most striking finding was a significantly shorter average hospital stay in the mebendazole group (7 days) compared with the control group (10 days, *p* < 0.001), reflecting quicker clinical stabilization. In terms of symptom resolution, the mebendazole group demonstrated earlier alleviation of key COVID-19 symptoms, including fever, dry cough, fatigue, and dyspnea. Fever subsided approximately 2–2.5 days earlier in the treatment group, while cough and shortness of breath improved 2–3 days faster than in the SOC group. These clinical improvements were supported by reductions in inflammatory markers such as C-reactive protein (CRP), suggesting a dual antiviral and anti-inflammatory mechanism of action. The study also noted better oxygenation parameters and fewer cases of progression to severe respiratory compromise in the mebendazole arm. Although promising, the study did not provide detailed subgroup analyses (e.g., age, comorbidities), and its findings are yet to be confirmed in multicenter trials. Nonetheless, this trial strengthens the case for further exploration of mebendazole as a low-cost, repurposed therapeutic agent with multidimensional benefits in COVID-19 treatment.

El-Tanani et al. (Jordan) [31]: In a randomized, placebo-controlled clinical trial, El-Tanani et al. evaluated the effects of mebendazole on clinical symptoms in outpatients with mild to moderate COVID-19. Patients receiving mebendazole showed notably faster resolution of systemic symptoms, including fever, fatigue, and malaise, with significant clinical improvement observed as early as the third day of treatment. This symptomatic relief was accompanied by a significant reduction in inflammatory markers, as evidenced by lower CRP levels in the treatment group (2.03 ± 1.45) compared with a placebo (5.45 ± 3.95, *p* < 0.001). Although the study was limited in duration, these results suggest that mebendazole may contribute to early clinical improvement in COVID-19 through a combination of symptom control and modulation of the inflammatory response. Further large-scale studies are needed to confirm these findings and explore long-term outcomes.

#### 2.2.3. COVID-19 Hospitalization and Mortality

Hospitalization rates and mortality were critical outcomes in the meta-analysis, particularly for assessing the potential of these drugs to prevent severe disease.

Vallejos et al. (Argentina) [39]: In this randomized trial assessing the effect of ivermectin in COVID-19 outpatients, hospitalization occurred in 5.6% (14/250) of participants in the ivermectin group compared with 8.4% (21/251) in the placebo group. Although this represented a lower hospitalization rate in the treatment group, the difference was not statistically significant (odds ratio 0.65; 95% CI: 0.32–1.31; *p* = 0.227). The mean time to hospitalization did not differ between groups. Notably, among those who required invasive mechanical ventilatory support, the time from study enrollment to MVS was significantly shorter in the ivermectin group (5.25 ± 1.71 days) than in the placebo group (10 ± 2 days; *p* = 0.019). However, there were no significant differences between groups in other secondary outcomes, including viral clearance or adverse events. These findings do not support a conclusive benefit of ivermectin in preventing hospitalization or improving overall clinical outcomes.

Naggie et al. (USA) [43]: In this randomized controlled trial involving 1800 participants (mean age 48 years; 58.6% women), hospitalization or death occurred in 1.2% of participants in both the ivermectin and placebo groups (ten vs. nine events, respectively), resulting in a hazard ratio of 1.1 (95% credible interval [CrI], 0.4–2.6). These findings indicate no significant difference in the risk of hospitalization or death between the two groups. The most frequently reported serious adverse events were COVID-19 pneumonia, which occurred in five participants in the ivermectin group and seven in the placebo group, and venous thromboembolism, reported in one ivermectin-treated participant versus five in the placebo arm. Overall, the results do not demonstrate a reduction in hospitalization or mortality with ivermectin use in this outpatient COVID-19 population.

Galal et al. (Egypt) [42]: This randomized interventional study evaluated the effects of mebendazole in hospitalized patients with moderate COVID-19. Participants received either standard of care or mebendazole (100 mg twice daily for 3 days) alongside SOC, with clinical and laboratory outcomes monitored throughout hospitalization. Patients in the mebendazole group had significantly shorter hospital stays compared with those in the control group (7 vs. 10 days, *p* < 0.001). Clinical symptom resolution was faster in the treatment group, with earlier improvements in fever (by approximately 2.5 days), cough, fatigue, and dyspnea. These improvements were paralleled by reductions in C-reactive protein (CRP), indicating a decrease in systemic inflammation. Additionally, patients in the mebendazole arm demonstrated improved oxygenation status, requiring fewer respiratory interventions, although this was not statistically quantified in the study. With regard to more severe outcomes, the mortality rate was numerically lower in the mebendazole group (4.4%) compared with the SOC group (9%), but this difference did not reach statistical significance (*p* = 0.35). As such, no definitive conclusions can be drawn regarding mortality benefit. The study was not powered to assess mortality or critical care outcomes, and the lack of statistical significance in these endpoints limit interpretation. Nonetheless, the findings related to symptom duration and inflammatory markers provide a rationale for further investigation in larger trials.

#### 2.2.4. Safety and Tolerability

Both ivermectin and mebendazole were well-tolerated in the included studies, with no significant increase in adverse events compared with a placebo or SOC.

Mohan et al. (India) [33]: From a qualitative perspective, the adverse event profiles of ivermectin at both 12 mg and 24 mg doses were similar to those observed in the placebo group, with no indication of dose-related toxicity. The absence of serious adverse events across all groups reinforces the general tolerability of ivermectin in a COVID-19 treatment context. Participants commonly reported mild gastrointestinal complaints, with epigastric burning sensation being the most frequent symptom, affecting roughly one in ten patients. Notably, this symptom was evenly distributed across treatment arms and did not correlate with drug dosage, suggesting it may be attributable to COVID-19 itself or nonspecific factors rather than the intervention. The similarity in adverse event rates between ivermectin and placebo groups highlights the safety of ivermectin in the studied doses and supports its continued evaluation in larger trials where safety remains a priority consideration alongside efficacy outcomes.

de la Rocha et al. (Mexico) [30]: The adverse event profile of ivermectin appeared largely comparable to that of the placebo group during the course of COVID-19 treatment. Although a total of 13 adverse events were reported in the ivermectin group, these were primarily attributed to the viral illness itself, mirroring the 17 events observed in the placebo group. Notably, only one serious adverse event—encephalitis—was reported in the ivermectin arm, but this was attributed to SARS-CoV-2 infection rather than the drug, and the patient fully recovered, underscoring the event’s likely disease-related etiology rather than treatment-induced toxicity. Clinical symptoms such as cough remained the most commonly reported across both arms, with no significant group-wise differences in symptom frequency. Furthermore, post-treatment laboratory evaluations revealed modest changes in cholesterol, platelet, and erythrocyte levels, with the latter showing a significant increase in the ivermectin group. These hematologic shifts, while statistically notable, were not accompanied by clinical deterioration. Overall, ivermectin was well tolerated and did not lead to an increased risk of adverse or severe events when compared with a placebo.

Mikamo et al. (Japan and Thailand) [47]: This trial provides strong evidence supporting the safety and tolerability of ivermectin in patients with mild COVID-19, including adolescents aged 12 years and older. The proportion of participants reporting adverse events was nearly identical between the ivermectin and placebo groups, suggesting that ivermectin does not contribute to increased adverse outcomes when compared with standard care. The adverse events most commonly reported were related to COVID-19 illness itself, such as pneumonia, rather than to the investigational product. Discontinuations due to adverse events were few—four in the ivermectin group and one in the placebo group—and were primarily driven by COVID-19 disease progression. Serious adverse events were rare and nonfatal, with urticaria being the only event considered treatment-related, occurring in a single placebo recipient. Importantly, no new safety concerns were identified in the subgroup of participants under 18, supporting ivermectin’s acceptable safety profile across age groups. Overall, the qualitative data affirm that ivermectin is generally well tolerated and does not elevate the risk of adverse or serious adverse effects in the context of COVID-19 management.

Hayward et al. (UK) [46]: Participants randomized to ivermectin experienced no meaningful increase in the frequency or severity of adverse events when compared with those receiving usual care. With only three serious adverse events in the ivermectin group versus five in the standard care group, the data reflect a low incidence of treatment-related complications. Moreover, long-term outcomes at 3, 6, and 12 months—including the proportion of participants feeling fully recovered—were similar between groups, suggesting no delayed safety concerns. This consistency in safety outcomes across both short- and long-term assessments supports the conclusion that ivermectin was well tolerated in a broad, community-based COVID-19 population. However, the absence of efficacy combined with the lack of any safety advantage led the investigators to recommend against further trials of ivermectin in similar settings.

Naggie et al. (USA) [43]: The safety profile of ivermectin was consistent with that of a placebo in this trial, involving a largely vaccinated population with mild COVID-19. The proportion of participants experiencing serious adverse events, including hospitalization or death, was identical (1.2%) in both treatment arms, and the types of serious events—primarily COVID-19 pneumonia and thromboembolic events—reflected complications of the disease rather than treatment-related toxicity. Notably, the incidence of venous thromboembolism was higher in the placebo group, although not statistically significant, and no new safety signals were identified for ivermectin. These findings suggest that ivermectin was well tolerated, with an adverse event profile indistinguishable from that of a placebo, reinforcing its short-term safety but not supporting its efficacy in altering disease trajectory.

Schilling et al. (Thailand) [34]: This study demonstrated that high-dose ivermectin was largely well tolerated in patients with early symptomatic COVID-19, with no serious adverse events attributed to the drug. All three serious adverse events occurred in the no-treatment arm and were linked to complications of COVID-19 itself rather than treatment interventions. In the ivermectin group, the most notable side effect was transient visual disturbance, reported by six patients. Although not severe by grading criteria, these visual changes prompted treatment discontinuation in three cases. Importantly, all symptoms resolved rapidly upon cessation of the drug, and ophthalmologic evaluation confirmed no residual visual abnormalities. This highlights the importance of monitoring even mild neurological or sensory symptoms during high-dose regimens. Overall, ivermectin demonstrated a favorable safety profile under clinical supervision, with no dose-limiting toxicity, though attention to rare, reversible visual disturbances is warranted in future trials or clinical use.

Galal et al. (Egypt) [42]: The safety profile of mebendazole in this study was favorable, with no patients requiring discontinuation of treatment due to adverse effects. Despite known risks associated with higher doses—such as gastrointestinal upset, hematologic abnormalities, and rare neurotoxic events—none of these were reported in the study population, suggesting good tolerability at the administered dose. Observed symptoms were mild and aligned with previously documented effects of the drug, primarily involving the gastrointestinal system. The lack of serious or dose-limiting toxicity, especially in a population that included both hospitalized and non-hospitalized patients, reinforces the short-term safety of mebendazole in COVID-19 management. However, the authors acknowledge demographic imbalances—such as younger median age in the outpatient treatment group and variations in baseline CRP and renal function in the inpatient cohort—that could confound interpretation of outcomes and perceived safety. These findings, while encouraging, highlight the need for prospective studies with controlled conditions and standardized adverse event reporting to confirm the safety and tolerability of mebendazole in diverse COVID-19 populations.

#### 2.2.5. Summary of Qualitative Findings

The qualitative findings from various studies on ivermectin and mebendazole present mixed evidence regarding their effectiveness in COVID-19 treatment, particularly in virological clearance, symptom resolution, hospitalization rates, and overall safety. Some studies have suggested that ivermectin may accelerate viral clearance and increase the likelihood of achieving a negative RT–PCR test earlier than placebo. However, others have failed to replicate these findings, indicating variability in its antiviral effects across different populations. Similarly, mebendazole has shown promise in increasing cycle threshold values, suggesting reduced viral load in treated patients. Despite these encouraging findings, the limited number of studies prevents definitive conclusions about its antiviral properties, highlighting the need for further research.

Clinical outcomes related to symptom resolution and hospital stays have also demonstrated inconsistent results. Some studies indicate that ivermectin can shorten the duration of symptoms such as cough and dyspnea, as well as reduce hospital stay, while others have found no significant benefits compared with standard-of-care treatments. Mebendazole has also been investigated in this context, with some studies reporting superior outcomes in symptom resolution and hospital stay reduction compared with ivermectin. However, these findings are not yet well established, as variations in study design, patient selection, and treatment protocols could influence the observed effects. More robust, large-scale trials with standardized assessment criteria are required to clarify the clinical utility of these drugs.

Hospitalization rates and mortality outcomes further add to the uncertainty surrounding the efficacy of ivermectin and mebendazole. While some studies have suggested a potential drift toward lower mortality with mebendazole, no strong statistical evidence supports its widespread use for preventing severe disease progression. Similarly, ivermectin has not demonstrated a consistent benefit in reducing hospitalization rates or mortality, raising concerns about its overall impact on COVID-19 outcomes. These findings underscore the importance of well-structured clinical trials to determine whether either drug has a meaningful role in managing severe cases of COVID-19.

Both ivermectin and mebendazole appear to have a favorable safety profile, with studies reporting minimal adverse effects even at higher doses. This suggests that these drugs could be considered for further investigation as potential therapeutic options. However, given the inconsistent findings on their efficacy, future research should focus on optimizing treatment regimens, identifying patient populations that may benefit the most, and exploring potential synergistic effects with other antiviral or immunomodulatory therapies. Standardizing study methodologies and endpoints will be crucial in determining the true therapeutic value of ivermectin and mebendazole in COVID-19 management.

## 3. Discussion

This meta-analysis provides a thorough evaluation of the potential role of anthelmintic drugs, specifically ivermectin and mebendazole, in the treatment of COVID-19. Given the urgency of identifying effective therapeutic interventions during the pandemic, several randomized controlled trials (RCTs) have investigated the efficacy and safety of these repurposed agents. While some studies suggest benefits in terms of viral clearance, symptom resolution, and hospitalization duration, the overall evidence remains inconclusive due to inconsistencies across trials. This discussion explores the implications of the findings, the strengths and limitations of the included studies, and future research directions.

While previous systematic reviews, including the comprehensive analysis by Popp et al. [49], have evaluated the efficacy and safety of ivermectin in COVID-19, they primarily concluded there was insufficient evidence to support its routine use. They emphasized the low certainty of evidence and recommended against routine use of ivermectin outside clinical trials due to concerns over bias, small sample sizes, and methodological weaknesses in the included studies. Similarly, Roman et al. (2021) raised significant concerns regarding the quality of evidence and inconsistent reporting of outcomes, ultimately concluding that available data did not support a definitive therapeutic role for ivermectin [50]. Conversely, some early meta-analyses, including those by Bryant et al. and Lawrie et al., suggested a mortality benefit and improved clinical outcomes with ivermectin, although these findings were later critiqued due to the inclusion of retracted or low-quality studies [51,52].

Compared with these previous reviews, our meta-analysis updates the evidence base by incorporating a broader set of recent and rigorously conducted randomized controlled trials (RCTs), applying a comprehensive Cochrane Risk of Bias 2.0 assessment, and evaluating both efficacy and safety outcomes. Importantly, our study not only distinguishes between the effects of ivermectin and mebendazole but also includes a more expansive set of clinical endpoints—including symptom duration, hospitalization time, and adverse events—thereby providing a multidimensional analysis of therapeutic impact. This integrative approach enhances the interpretability and applicability of our findings in both clinical and public health contexts. Furthermore, the exploration of underlying immunomodulatory mechanisms offers a novel contribution, supporting biological plausibility and enriching the clinical relevance of the observed outcomes.

However, this meta-analysis expands upon prior work by incorporating newly available randomized controlled trials and exploring not only antiviral efficacy but also the mechanistic immunomodulatory and anti-inflammatory roles of ivermectin and mebendazole. A cogent rationale for continued investigation lies in their unique pharmacodynamic properties and potential utility in resource-constrained settings. Both drugs are inexpensive, widely available, and have favorable safety profiles, making them attractive candidates for low- and middle-income countries where access to advanced therapies such as monoclonal antibodies or novel antivirals may be limited. Moreover, the therapeutic window of these drugs allows for repurposing at relatively low doses, potentially reducing the burden on healthcare systems. Mebendazole’s emerging profile as an mTOR and tubulin polymerization inhibitor with immunoregulatory benefits adds novelty beyond traditional antiviral paradigms. Investigating these agents in rigorously designed, large-scale, multicenter trials could yield scalable treatment strategies that are both clinically impactful and economically viable. Thus, despite some overlap with earlier findings, this study contributes new mechanistic insights and a public health perspective that justify further research, particularly in global contexts where cost-effective therapeutics are urgently needed.

The analysis of ivermectin trials yielded conflicting results. Several studies, such as those conducted by Ahmed et al. (Bangladesh) and Biber et al. (Israel), suggested that ivermectin may accelerate viral clearance and improve clinical outcomes [28,41]. These studies reported a reduction in time to negative RT–PCR results and symptomatic relief, particularly when the drug was administered early in the disease course. Mechanistically, ivermectin is believed to exert immunomodulatory and anti-inflammatory effects by inhibiting the translocation of viral proteins into the host nucleus via importin α/β1 pathways, thereby disrupting viral replication. Furthermore, ivermectin modulates host immunity by downregulating nuclear factor kappa B (NF-κB) signaling and reducing levels of key pro-inflammatory cytokines such as IL-6 and TNF-α, which are implicated in the cytokine storm observed in severe COVID-19 cases. It also enhances the production of interferon-γ and increases macrophage phagocytic activity, contributing to antiviral defense. Ivermectin may promote a shift from a pro-inflammatory Th1-type immune response to a more regulated immune state, potentially mitigating the immunopathology that leads to acute respiratory distress syndrome (ARDS). These pleiotropic immunological actions suggest that ivermectin may act synergistically with corticosteroids, IL-6 inhibitors (e.g., tocilizumab), and Janus kinase (JAK) inhibitors by amplifying anti-inflammatory signaling or minimizing cytokine spillover [53,54].

However, large-scale RCTs, including those conducted by Mikamo et al. (Japan & Thailand) and Hayward et al. (UK), found no significant difference between ivermectin and placebo regarding viral clearance, hospitalization rates, or symptom resolution [46,47]. The inconsistencies in findings may be attributed to several factors, including differences in dosing regimens, disease severity at initiation, variations in patient demographics, and study design heterogeneity. Notably, ivermectin trials exhibited a high degree of heterogeneity (I^2^ = 82% for viral clearance and I^2^ = 100% for hospitalization duration), reflecting variations in study protocols and patient characteristics. Some studies employed single-dose regimens, while others used multiple-dose regimens over varying durations. Such inconsistencies complicate the interpretation of ivermectin’s true efficacy in COVID-19 management.

Compared with ivermectin, mebendazole was studied in fewer trials, yet its reported benefits appeared more consistent. Studies such as those conducted by Galal et al. (Egypt) and El-Tanani et al. (Jordan) indicated that mebendazole significantly reduced viral load, systemic inflammation, and hospitalization rates [31,42]. Mebendazole’s immunomodulatory and anti-inflammatory actions are attributed to its ability to inhibit tubulin polymerization, disrupting microtubule-dependent functions essential for viral replication. Additionally, it has been shown to suppress the production of pro-inflammatory mediators, including IL-1β, TNF-α, and nitric oxide synthase (NOS), thereby attenuating tissue inflammation. Mebendazole has been demonstrated to activate AMP-activated protein kinase (AMPK) and inhibit mammalian target of rapamycin (mTOR), pathways known to modulate autophagy and inflammation. Moreover, it modulates immune cell function by reducing neutrophil infiltration and dampening oxidative stress while promoting the expansion of regulatory T cells (Tregs), which help resolve inflammation and maintain immune tolerance. This broad spectrum of immunomodulatory effects positions mebendazole as a promising adjunct in combination therapies with antivirals such as remdesivir, or with immunosuppressive agents like corticosteroids or calcineurin inhibitors, particularly in hyperinflammatory stages of COVID-19 [16,55].

The pooled analysis suggested a slight increase in RT–PCR cycle threshold (Ct) values among patients treated with ivermectin or mebendazole, indicating a potential reduction in viral load. However, this effect was not statistically significant (*p* = 0.39), and substantial heterogeneity was observed across studies (I^2^ = 82%). While some individual trials reported a positive tendency toward improved viral clearance, others found minimal or no impact. Factors contributing to these inconsistencies include variability in treatment protocols (dose and duration), differences in baseline disease severity, timing of post-treatment viral load assessment, and study population differences, including age and comorbidities. Future research should focus on subgroup analyses to determine whether specific patient populations may benefit more from ivermectin or mebendazole in enhancing viral clearance.

The analysis of hospitalization duration yielded mixed findings. While some studies demonstrated a significant reduction in hospital stay with ivermectin or mebendazole, others reported negligible or even opposing effects. The pooled effect size suggested a potential reduction in hospitalization time (Cohen’s d = −0.76, *p* = 0.15), yet the high level of heterogeneity (I^2^ = 100%) complicates interpretation. Potential reasons for these discrepancies include differences in COVID-19 severity at enrollment (mild vs. severe cases), variations in healthcare systems and hospital discharge criteria, and inconsistent definitions of hospitalization duration across studies.

Analysis of symptom resolution time showed an inclination toward faster recovery in patients receiving ivermectin or mebendazole (Cohen’s d = −0.84, *p* = 0.08), but the effect did not reach statistical significance. Some studies, such as those by Shahbaznejad et al. and Hayward et al., reported notable reductions in symptom duration, whereas others found minimal or no difference between treatment and placebo groups [45,46]. Given the extreme heterogeneity observed (I^2^ = 99%), it is essential to conduct further stratified analyses based on disease severity, comorbidities, and symptom-specific responses to treatment.

Both ivermectin and mebendazole were well tolerated in the included studies. No significant increase in adverse events was reported compared with a placebo or standard of care (SOC). High-dose ivermectin (up to 84 mg) was found to be safe in trials such as Buonfrate et al., although no corresponding improvement in viral clearance was observed [29]. Similarly, mebendazole demonstrated an acceptable safety profile, with no significant treatment-related adverse effects reported [31,42]. The favorable safety profiles of both drugs suggest that they could be further explored as potential therapeutic options. However, given the inconsistent findings regarding efficacy, more rigorous investigations are needed to establish their clinical utility in COVID-19 treatment.

Moreover, studies should investigate the synergistic potential of ivermectin and mebendazole when used in combination with other antiviral, immunomodulatory, or anti-inflammatory agents, particularly in patients with hyperinflammatory responses or cytokine storm syndromes. Such combination therapies may offer enhanced clinical outcomes through complementary mechanisms of action. For instance, combining ivermectin’s inhibition of nuclear transport and suppression of NF-κB with mebendazole’s inhibition of mTOR and promotion of autophagy could provide a two-pronged approach to reducing inflammation while inhibiting viral replication. Additionally, the pairing of these drugs with immune checkpoint inhibitors or monoclonal antibodies could be explored in high-risk COVID-19 cohorts to potentiate adaptive immune responses while minimizing systemic inflammation. This rationale supports future studies designed with factorial or adaptive trial designs to identify effective multidrug regimens tailored to disease stages and patient phenotypes.

While our findings are generally consistent with the cautious interpretations of large-scale reviews like that of Popp et al. [49], this analysis goes a step further by quantifying safety and tolerability outcomes through a dedicated meta-analysis, which many earlier studies only described narratively or excluded altogether. Additionally, by including trials evaluating mebendazole—a promising yet understudied repurposed agent—this study extends the conversation beyond ivermectin, allowing for a more comparative evaluation of repurposed anthelmintics in COVID-19 treatment. The inclusion of forest plots specific to adverse events, along with detailed mechanistic rationales, helps to position these drugs within the broader therapeutic landscape of COVID-19, especially for resource-limited settings where access to novel antivirals may be constrained. This updated and expanded evidence base enables a more informed perspective on the nuanced benefits and limitations of repurposed antiparasitic agents while reinforcing the need for high-quality, multicenter, and standardized clinical trials to validate potential therapeutic roles.

This meta-analysis provides a comprehensive synthesis of randomized controlled trials evaluating the safety and efficacy of ivermectin and mebendazole in the treatment of COVID-19, incorporating data from diverse geographical settings and patient populations. The use of a rigorous search strategy, adherence to PRISMA guidelines, and application of the Cochrane RoB 2.0 tool for bias assessment strengthen the reliability of our findings. However, the study also has certain limitations. Notably, there was an imbalance in the number of included studies—only two trials assessed mebendazole, while the majority focused on ivermectin—limiting comparative conclusions. Furthermore, significant heterogeneity across trials in terms of patient demographics, baseline comorbidities, disease severity, and treatment protocols, as well as the inconsistent reporting of stratified outcomes, constrained our ability to perform subgroup analyses. These factors should be considered when interpreting the pooled results and highlight the need for more uniform and high-quality future trials.

## 4. Materials and Methods

### 4.1. Search Strategy

A comprehensive and systematic literature search was conducted across four major electronic databases—Scopus, PubMed, Embase, and the Cochrane Library—to identify relevant studies published up to February 2025. The search was designed following the PRISMA (Preferred Reporting Items for Systematic Reviews and Meta-Analyses) guidelines to ensure methodological rigor. The focus was on randomized controlled trials (RCTs) evaluating the efficacy of ivermectin and mebendazole in treating COVID-19 patients compared with a placebo or standard of care (SOC). This approach ensured the inclusion of high-quality, peer-reviewed evidence for the meta-analysis.

To construct a robust and reproducible search strategy, a combination of Medical Subject Headings (MeSH) terms, keywords, and Boolean operators (AND, OR, NOT) was employed. The primary search terms included disease-related keywords (“COVID-19” OR “SARS-CoV-2” OR “coronavirus disease 2019”) combined with intervention-related terms (“Ivermectin” OR “Mebendazole” OR “anthelmintic drugs”) and study design filters (“randomized controlled trial” OR “RCT” OR “clinical trial”). To ensure a precise and targeted selection of studies, we applied the AND operator to retrieve only those studies that included both terms, COVID-19 AND (Ivermectin OR Mebendazole), in the title. Furthermore, relevant outcome terms were incorporated (“virological clearance” OR “symptom resolution” OR “hospitalization” OR “mortality”) to ensure a targeted selection of studies assessing key clinical endpoints. The search also included comparison terms such as “placebo” OR “standard of care” OR “SOC” to specifically identify trials with controlled designs. Additionally, non-relevant study types, including “case reports” OR “observational study” OR “preprint”, were excluded to enhance the reliability of the findings.

The inclusion criteria focused on selecting randomized controlled trials (RCTs) comparing ivermectin or mebendazole to a placebo or standard of care in adult patients (≥18 years) with confirmed COVID-19. Eligible studies were required to report at least one of the primary clinical outcomes, namely virological clearance, symptom resolution, hospitalization, or mortality. Furthermore, only studies published in English were considered to maintain consistency in data interpretation. Studies that did not meet these criteria were excluded from the meta-analysis to uphold methodological precision.

Exclusion criteria were applied to filter out studies that did not meet the predefined methodological standards. Non-randomized studies, case reports, observational studies, and studies without a control group were excluded due to the higher risk of bias and lack of causal inference. Additionally, studies with insufficient data for meta-analysis were omitted to ensure the robustness of statistical synthesis. Preprints and non-peer-reviewed articles were also excluded to prioritize peer-reviewed evidence, minimizing the inclusion of potentially unreliable or non-validated findings.

### 4.2. Data Extraction and Quality Assessment

Following the search and selection process, three independent reviewers screened and extracted data from the eligible studies using a standardized extraction form. Key variables included study ID, author, publication year, geographic location, sample size, intervention details (dose and duration), and reported outcomes. To evaluate the methodological quality and internal validity of the studies included in this meta-analysis, a comprehensive risk of bias assessment was conducted using the Cochrane Risk of Bias 2.0 (RoB 2.0) tool. This framework assesses potential sources of bias across five key domains: the randomization process, deviations from intended interventions, missing outcome data, measurement of outcomes, and selection of reported results.

A total of 23 randomized controlled trials (RCTs) investigating the efficacy and safety of ivermectin and mebendazole in the treatment of COVID-19 were systematically evaluated. The findings are summarized as follows (Supplementary Table S2).

#### 4.2.1. Randomization Process

All included studies employed robust methods for random sequence generation, most commonly utilizing computer-based randomization tools. Additionally, allocation concealment was appropriately maintained in nearly all trials through mechanisms such as sealed opaque envelopes, central randomization services, or third-party allocation systems. These measures significantly reduce the risk of selection bias.

#### 4.2.2. Deviations from Intended Interventions

The majority of trials ensured adequate blinding of both participants and personnel, which mitigates the risk of performance and detection bias. Outcome assessors were also blinded in most cases. Importantly, there were no major protocol deviations or violations reported that would compromise the fidelity of the interventions being evaluated.

#### 4.2.3. Missing Outcome Data

Most studies reported complete or near-complete outcome data, with low rates of attrition that were balanced across study arms. Where dropouts occurred, the reasons were well explained and were not associated with treatment assignment or adverse outcomes. Intention-to-treat analyses were used consistently, further minimizing bias.

#### 4.2.4. Measurement of Outcomes

All trials utilized objective, clinically relevant outcome measures, including RT–PCR-confirmed viral clearance, time to symptom resolution, hospitalization rates, and mortality. Standardized tools and definitions were employed to ensure consistency across study sites and populations, enhancing the reliability of outcome assessments.

#### 4.2.5. Selection of the Reported Results

Nearly all studies had pre-registered protocols or were listed in trial registries such as ClinicalTrials.gov. Outcomes reported in the final publications were generally consistent with those prespecified, minimizing the risk of selective outcome reporting. In several trials, supplementary data and statistical analysis plans were made available, further improving transparency.

Based on these criteria, the overall risk of bias was determined to be low for the majority of the included RCTs. This consistent methodological rigor across studies strengthens the credibility of the evidence base and supports the validity of the pooled effect estimates derived from this meta-analysis. Although a few studies exhibited minor methodological limitations in one or two domains, these were not judged to materially affect the overall risk profile or the direction of results. This rigorous quality assessment ensured that only high-quality studies were included, strengthening the validity and reliability of the meta-analysis.

### 4.3. Statistical Analysis

Quantitative data were analyzed using IBM SPSS Statistics (Version 29; IBM Corp., Armonk, NY, USA). Descriptive statistics were used to summarize the data, while inferential statistical tests were applied to determine the significance of the findings. Heterogeneity across studies was assessed using the I^2^ statistic, and a random-effects model was employed when substantial heterogeneity was detected. Subgroup analyses and sensitivity analyses were conducted to explore potential sources of variability. Statistical significance was set at *p* < 0.05. The results were presented as pooled effect estimates with 95% confidence intervals (CIs), ensuring a robust meta-analytical approach.

## 5. Conclusions

The COVID-19 pandemic has underscored the need for repurposing existing drugs to identify cost-effective and widely accessible treatments. This meta-analysis provides valuable insights into the potential role of anthelmintic drugs, particularly ivermectin and mebendazole, in the management of COVID-19. While ivermectin demonstrated some benefits in specific studies, the overall evidence remains inconclusive, warranting further research to establish its optimal dosing and patient selection criteria. Mebendazole, though less studied, showed promising results in reducing viral load and inflammatory markers, making it a potential candidate for further investigation.

Despite these promising findings, the current evidence does not support the widespread clinical use of ivermectin or mebendazole for COVID-19 outside of well-designed clinical trials. Future research should focus on large-scale, multicenter RCTs with standardized dosing regimens and well-defined patient subgroups to clarify the efficacy of these drugs. Mechanistic studies exploring their antiviral and immunomodulatory effects could further enhance our understanding of their therapeutic potential.

From a clinical and policy perspective, while ivermectin and mebendazole may offer an affordable option in resource-limited settings, their use should be guided by clinical judgment and evidence-based guidelines. Policymakers should prioritize funding for further research to validate these findings, ensuring that any potential benefits are realized in a safe and effective manner.

In summary, this meta-analysis highlights the complexities of repurposing anthelmintic drugs for COVID-19 treatment. While preliminary data suggest potential benefits, particularly for mebendazole, substantial evidence gaps remain. Addressing these gaps through well-structured clinical trials will be crucial in determining whether these drugs can play a meaningful role in the ongoing fight against COVID-19.

## Data Availability

All data arising from this study are included within the article.

## Supplementary Materials

The following supporting information can be downloaded at: https://www.mdpi.com/article/10.3390/antibiotics14050459/s1, Table S1: Characteristics of the included studies; Table S2: Risk of bias.
