# Supplementary material for: Repurposing Anthelmintic Drugs for COVID-19 Treatment: A Comprehensive Meta-Analysis of Randomized Clinical Trials on Ivermectin and Mebendazole
Source: Antibiotics (Basel). 2025 Apr 30;14(5):459. doi: 10.3390/antibiotics14050459 (PMC12108154; doi:10.3390/antibiotics14050459)
Supplement: Supplementary file 1 [file antibiotics-14-00459-s001.zip › Supplementary Table S2-Risk of bias.pdf]

Risk of Bias Table (RoB 2.0): Assessment for Included RCTs

Study (Author, Year)	Randomization Process	Deviations from Interventions	Missing Outcome Data	Measurement of Outcomes	Selection of Reported Results	Overall RoB
Ahmed et al., 2021	Low	Low	Low	Low	Low	Low
Schilling et al., 2023	Low	Low	Low	Low	Low	Low
Wijewickrema et al., 2024	Low	Low	Low	Low	Low	Low
Chowdhury et al., 2021	Low	Low	Low	Low	Low	Low
Krolewiecki et al., 2021	Low	Low	Low	Low	Low	Low
Elgazzar et al., 2021	Low	Low	Low	Low	Low	Low
Mohan et al., 2021	Low	Low	Low	Low	Low	Low
Hashim et al., 2021	Low	Low	Low	Low	Low	Low
Okumus et al., 2021	Low	Low	Low	Low	Low	Low
Niaee et al., 2021	Low	Low	Low	Low	Low	Low
Shouman et al., 2021	Low	Low	Low	Low	Low	Low
Samaha et al., 2021	Low	Low	Low	Low	Low	Low
Babalola et al., 2021	Low	Low	Low	Low	Low	Low
Kirti et al., 2021	Low	Low	Low	Low	Low	Low
Pott-Junior et al., 2021	Low	Low	Low	Low	Low	Low
Chaccour et al., 2021	Low	Low	Low	Low	Low	Low
Mahmud et al., 2021	Low	Low	Low	Low	Low	Low
Gonzalez et al., 2021	Low	Low	Low	Low	Low	Low
López-Medina et al., 2021	Low	Low	Low	Low	Low	Low
Ravikirti et al., 2021	Low	Low	Low	Low	Low	Low
Beltrán González et al., 2021	Low	Low	Low	Low	Low	Low
Vallejos et al., 2021	Low	Low	Low	Low	Low	Low
Carvallo et al., 2020	Low	Low	Low	Low	Low	Low
